# Using Human-Centered Design to Build a Digital Health Advisor for Patients With Complex Needs: Persona and Prototype Development

**DOI:** 10.2196/10318

**Published:** 2019-05-09

**Authors:** Onil Bhattacharyya, Kathryn Mossman, Lovisa Gustafsson, Eric C Schneider

**Affiliations:** 1 Institute for Health Systems Solutions and Virtual Care Women's College Hospital University of Toronto Toronto, ON Canada; 2 Department of Family and Community Medicine University of Toronto Toronto, ON Canada; 3 Commonwealth Fund New York City, NY United States

**Keywords:** chronic disease, user-centered design, medical applications

## Abstract

**Background:**

Twenty years ago, a “Guardian Angel” or comprehensive digital health advisor was proposed to empower patients to better manage their own health. This is now technically feasible, but most digital applications have narrow functions and target the relatively healthy, with few designed for those with the greatest needs.

**Objective:**

The goal of the research was to identify unmet needs and key features of a general digital health advisor for frail elderly and people with multiple chronic conditions and their caregivers.

**Methods:**

In-depth interviews were used to develop personas and use cases, and iterative feedback from participants informed the creation of a low-fidelity prototype of a digital health advisor. Results were shared with developers, investors, regulators, and health system leaders for suggestions on how this could be developed and disseminated.

**Results:**

Patients highlighted the following goals: “live my life,” “love my life,” “manage my health,” and “feel understood.” Patients and caregivers reported interest in four functions to address these goals: tracking and insights, advice and information, providing a holistic picture of the patient, and coordination and communication. Experts and system stakeholders felt the prototype was technically feasible, and that while health care delivery organizations could help disseminate such a tool, it should be done in partnership with consumer-focused organizations.

**Conclusions:**

This study describes the key features of a comprehensive digital health advisor, but to spur its development, we need to clarify the business case and address the policy, organizational, and cultural barriers to creating tools that put patients and their goals at the center of the health system.

## Introduction

### Background

Twenty years ago, Peter Szolovits and colleagues proposed a digital “Guardian Angel” built on the notion that information systems designers might shift focus from serving health institutions to empowering the individual patient [1]. The Guardian Angel (or digital health advisor) would collect individual patient data, monitor health conditions, interpret health information for patients, help customize treatment plans, share information with the care team, and provide reminders and alerts about medications and appointments. With recent technological advances, the capacity to create a digital health advisor is now within reach, but it has yet to emerge. One barrier to the development of a digital health advisor might be the lack of a clear set of requirements to inform its design. Although there are a range of tools that perform some related functions and a few studies that cover some needs, they have not been presented in a way that is actionable for developers [2]. The Commonwealth Fund, a foundation whose goal is to improve access to health care, has been exploring this opportunity through its program on the information technology (IT)-enabled consumer. The fund has a particular interest in models of care for high-need, high-cost (HNHC) patients and ways to enhance coordination as well as patient and caregiver engagement [3]. We sought to determine how the concerns of HNHC patients and their caregivers might be met through digital tools in order to communicate this to the stakeholders who can create and distribute these tools. This paper describes this strategy, highlighting the use of persona development and an illustrative prototype to engage key stakeholders in order to promote the creation of a digital health advisor for the people who could derive the most benefit.

## Methods

### Solutions to Improve the Care of High-Need, High-Cost Patients

Information and communication technologies (ICTs) have transformed many industries, often by reconfiguring services around the needs of consumers. But health care has lagged behind. Many of the current consumer-facing applications target people who are relatively healthy [4]. There has been less focus on the 5% of the population who account for nearly 50% of all system costs in the United States and in other regions [5]. These HNHC individuals often have multiple chronic conditions and are more likely to be lower income and face housing and food insecurity [6-9]. They are poorly served by a health system built around diseases and institutions that often results in fragmented and competing care [10,11]. Among population segments, frail elderly are the most likely (46.2%) to be high cost, followed by adults under 65 years with disability and adults with major complex chronic disease [12]. They all have to engage in a variety of activities to improve their health [13,14], and digital technologies may be able to assist. As the largest subgroup, seniors are increasingly adopting technology, with 76% using cell phones, 64% using computers, 43% using the internet, and 40% using email and texting [15]. They are already engaging with some of these new approaches in health care: 16% of seniors search the internet to obtain health information, 8% to fill prescriptions, 7% to contact physicians, and 5% to handle insurance matters [15]. However, while there are more than 165,000 health apps currently available, a 2016 review found that most available digital tools for chronic disease are piecemeal, have limited functionality, and do not address the needs of patients with complex chronic conditions [4,16]. Furthermore, a review of evaluations of apps for conditions associated with higher needs found that most studies were small and few assessed process or outcome measures [17]. Another review compiled proposed functions from different studies and highlighted that tools should be developed with user needs in mind [2].

### Understanding and Communicating User Needs

Human-centered design (HCD) has been used to develop many transformative ICT solutions, is routine in a range of industries, and is increasingly applied in health care [10,18-27]. HCD is a problem-focused method that emerged from the fields of industrial design and (more recently) software development [28-30]. Strengths of design thinking include rapidly developing a deep understanding of user needs and then communicating them in ways that are emotionally engaging and actionable. This approach often involves interviews, observation, and immersion in a user’s context to develop user personas, or archetypes, and use cases [22,27]. A persona is a detailed description of a fictional person (often a composite of real individuals) used to communicate the key motivations, concerns, and interests of a user group [10,26,27]. Related to the persona is a use case or user story, which is a story with a plot describing the actions and decisions of a user in a particular context [26,27]. These representations can help designers foster empathy, better understand user needs, and develop new service options and tools [26]. Persona development has been used in a number of studies, but they have focused on specific chronic conditions (eg, older adults with diabetes [31], heart disease [32,33], or multiple sclerosis [34]) or groups like high users of the emergency department [35]. One study created personas of the “oldest old” but focused on implications for the design of computer interfaces [27]. None of these studies looked at a range of HNHC patients, and none were focused on designing a general tool to help achieve patient goals like the digital health advisor.

### Designing a Prototype for a Digital Health Advisor

The Commonwealth Fund was interested in developing a vision of a digital health advisor that could meet the needs of HNHC patients and using this to encourage its development in the health care marketplace by developers, entrepreneurs, investors, health system providers, funders, and regulators. The Fund worked with the design firm gravitytank to identify the needs of HNHC patients and their caregivers and create a low-fidelity prototype, which simulates key features of interest to this group without building out any of the functions [36,37]. This involved interviews with experts, 8 patient-caregiver pairs of people with multiple chronic conditions or frail elderly, and mapping the workflow of 3 care managers with similar patients [36,37]. Based on analysis of these data, a rough prototype of a digital health advisor was created and shared with patients and caregivers so they could provide feedback on the usability and functionality of the tool. This informed different outputs to make the insights more actionable.

## Results

**Figure 1:**
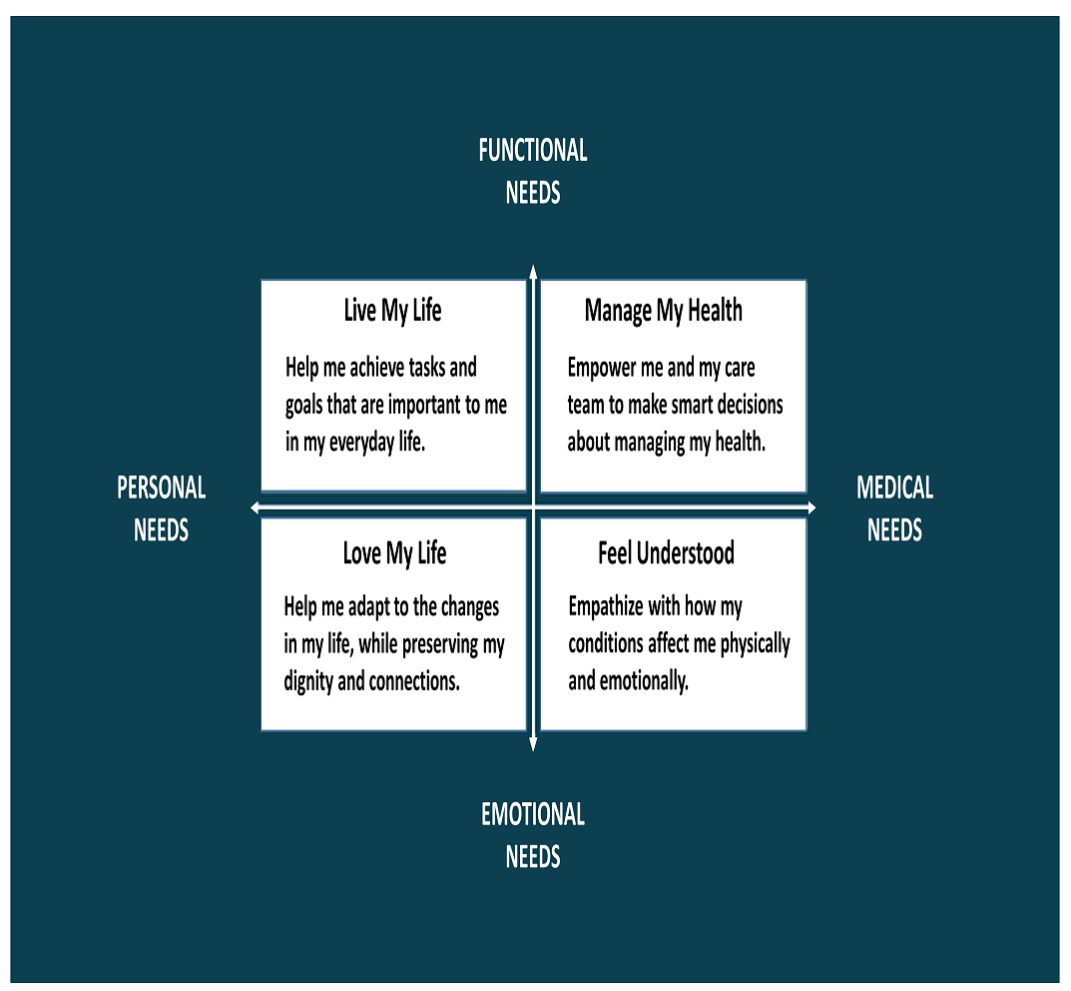
Key themes regarding patient needs.

**Figure 2:**
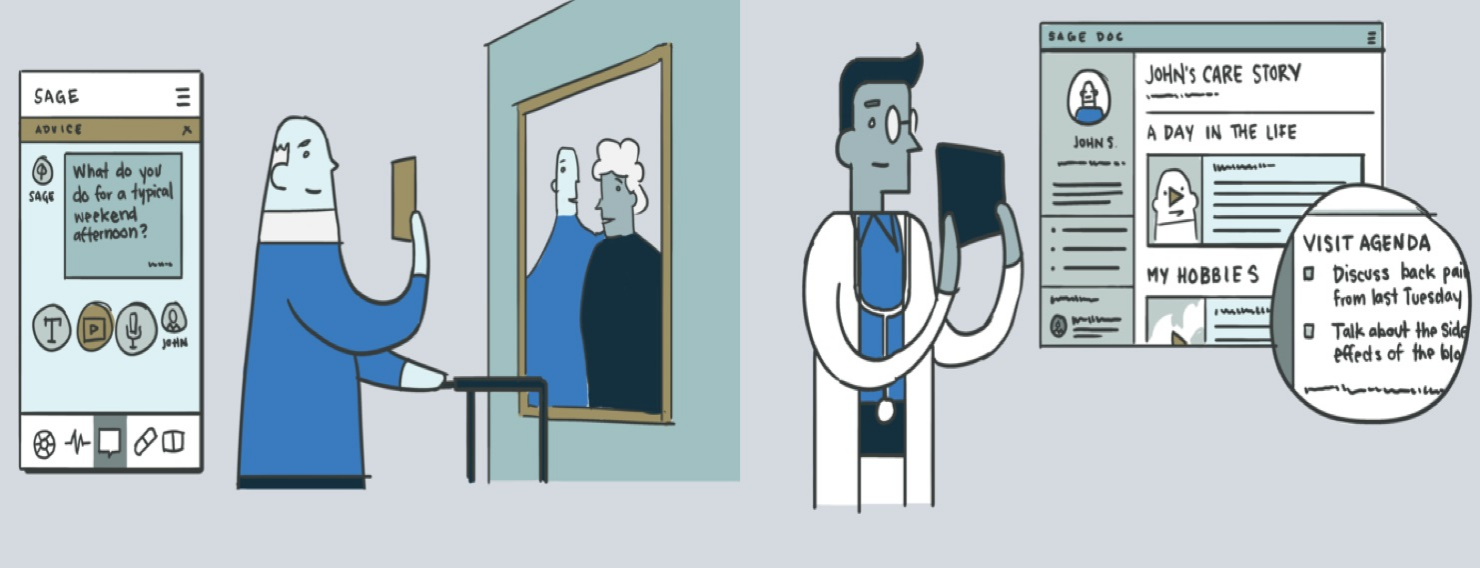
Sample use case for digital health advisor: John is an 87-year-old man living in San Mateo, California, with hypertension, arthritis, and mild cognitive impairment. He has been feeling more tired, anxious, and clumsy recently. He lives with his wife, who is his primary caregiver, and gets help from his daughter. He has an upcoming appointment with a geriatrician and he’s concerned whether this new doctor will understand what his needs are. He would like to update his doctors and highlight his activities and goals to share a more holistic picture of his life with the care team. He also wants to manage his doctor appointments and keep track of what his doctor tells him.

First, the needs of patients with multiple chronic conditions and the frail elderly and their caregivers were characterized, focusing on the intersection of functional and emotional needs with medical and personal needs (see Figure 1). Patients felt that a tool that addressed functional needs could improve health outcomes, but if a tool addressed emotional needs they would be more likely to use it on an ongoing basis. One link between the functional and emotional was having a tool that could connect goals to functions and orient care so that it can better manage their conditions in order to increase their ability to do the things that make life worthwhile. They highlighted the challenges of communicating with family members about end of life, sharing goals, navigating apps, and approval processes. They also had difficulty understanding the wide range of uncoordinated advice they receive and following through on the subset of recommendations that address their needs. Based on these interviews, 4 patient personas and 2 caregiver personas were developed (see Textbox 1) along with 4 use cases (see Figure 2 for an example). Last, a low-fidelity prototype of a digital health advisor was developed with a number of key features (see Textbox 2, with screenshots in Multimedia Appendix 1) [38].

**Overview of patient and caregiver personas.**

**Patient personas**

- “John”
  - An 87-year-old frail elderly man dealing with arthritis, hypertension, and mild cognitive impairment
  - He needs a tool to reach out to loved ones when he needs help, communicate his goals and current level of function, and give prompts for key health behaviors
- “Elizabeth”
  - A 70-year-old elderly patient with multiple chronic conditions
  - She needs help managing her medications, tracking her health status, and learning about activities to improve her health
- “Karen”
  - A 65-year-old patient with multiple chronic conditions
  - She needs help coordinating her care with her providers, managing her conditions, and accessing relevant health and social services
- “Jasmine”
  - A 44-year-old patient with a major chronic condition
  - She needs help communicating with her family about her health, managing ongoing symptoms, and learning about new treatments

**Caregiver personas**

- “Beth”
  - A 79-year-old caregiving spouse
  - She needs help managing her spouse’s medications and appointments and connecting with medical and community resources
- “Lisa”
  - A 45-year-old caregiver for her elderly mother
  - She needs help coordinating her mother’s care, remotely monitoring her health status, and communicating with family members regarding her mother’s health

**Key features of a digital health advisor for high-need, high-cost patients and their caregivers.**

**Tracking and insights:**

- Metrics dashboard collects health data such as blood pressure, oxygen levels, and gait from sensors and connected devices or receives manual input on symptoms
- Tracks current state before a medical visit or patterns over time, provides recommendations to patients, and informs the care team of changes in health status
- Highlights patient’s functional status and summarizes current symptom and risk factor control for different diseases so that care can focus on improving key functions

**Advice and information:**

- Personalized advice on health-related questions either through integration with a digital assistant or by connecting to a medical practitioner by text, voice, or video
- This advice would draw on information from medical records, personal metrics, insurance coverage, and available community resources
- The advice would either elicit or incorporate known preferences into recommendations

**Holistic picture:**

- A care journal and patient profile where patients can provide a brief image, a written summary of their story, and a set of goals and milestones they want to track
- This would include a checklist of care preferences to help create a comprehensive picture of their life to share with practitioners and foster deeper connections with their medical team and caregivers

**Coordination and communication:**

- A shared calendar with all appointments, a document center, and a task manager to improve coordination and communication between patients and their care team, including help with scheduling and transportation
- This would also include transparent communications between providers about a patient’s care to highlight areas of ambiguity and ensure agreement between members of the team

The personas and prototypes of the digital health advisor were presented to a group of policymakers; regulators; clinicians; experts in informatics, advertising, and health services; patient advocates; technology industry executives; and health system leaders. They felt that most components are technically feasible using current technology, although further development is needed in decision support. Patients and participants from the advertising industry highlighted the need for relatability of the tool and a “stickiness” that made it the first thing patients turn to when they have a question about their health. They highlighted the need for a simple visual interface and opportunities for voice activation to benefit those with low literacy or cognitive challenges. Health system leaders highlighted how a dashboard with trends and the current state could make visits more efficient for people with complex conditions, by reducing the time needed to collect this information, leaving more time to discuss how to advance particular goals of care. They also mentioned how a tool to share detailed but actionable information on goals, functional status, physiologic parameters, and current burden of treatment could facilitate challenging therapeutic decisions involving multiple providers. Representatives from health care delivery organizations were felt to be critical to the development of a digital health advisor but stressed that it would also need to foster support in target communities, including patients, caregivers, families, and social networks. They stated that extensive collaboration is needed to develop a robust digital health advisor, which should be an integrated suite of tools. They also highlighted that novel analytics are not sufficient; data must be made available in a usable format, and the necessary data for real-time decision making (eg, cost, quality, availability of services) are currently held by many different groups in different formats. They suggested a useful digital health advisor is more likely to emerge in response to partnerships with organizations focused on consumer needs, such as consumer advocacy groups and large retail companies. Business cases and policy incentives need to be developed to encourage broad data sharing among community, government, and commercial initiatives.

## Discussion

### Designing Tools and Systems That Will Meet Patient Needs

The concept of a comprehensive digital health advisor for patients, which was proposed more than 20 years ago, is now technically feasible and attractive to HNHC patients in the United States. The patients highlight a range of functional, emotional, medical, and personal needs that might be addressed by this tool. They also list a series of key functions such as tracking, advice, providing a holistic picture of themselves, and coordination and communication. The use of human-centered design helps overcome the limitation that new designs in health care are often dreamed up by providers to be easy to implement but not necessarily use by people who rarely use health services and don’t resemble the people they are trying to help. Personas can promote stakeholder engagement by providing detailed information on patient needs and how a tool might address those needs, making it easier to understand key functions, possible interactions with the health system, data requirements, and potential regulatory concerns.

While some studies have employed human-centered design to develop tools for patients with multiple chronic conditions [39,40], this approach tends to be applied to specific populations (eg, people with diabetes) [26,41,42] or specific uses (eg, communication, monitoring) [43,44]. Our work supplements the findings of a recent review of mobile technologies for older adults that summarized the following design features from different articles: graphs; notification systems; text and video messaging; scheduling; and vision, hearing, and memory aids [2]. Even though there are many digital health tools on the market, few comprehensively address the needs of HNHC patients and even those tend to focus on medically defined needs rather than act as general advisors. To go from an understanding of needs to a functioning tool that meets those needs in the context of someone’s life is a stretch and it involves testing different use cases, functions, and target groups before finding a good fit. The personas and prototype helped elicit specific advice from providers, payers, regulators, developers, and investors by making the problems and options more tangible. This highlighted the range of challenges with trust, uptake, integration with workflows, value proposition for different stakeholders, and potential business models, going beyond a narrow focus on end user needs and features of the technology.

The learnings from this project about current HNHC patients could be applied to vulnerable populations at risk of becoming HNHC patients (another target group for the Commonwealth Fund) and even the general public. We found the highest users of the health system are interested in tools that can help them manage their health challenges but even more in helping them live their lives to the fullest. This is likely to be true of all patients, who not only value disease management but also see health as a means to identify and work toward their life goals. An individual’s health is intertwined with their psychological, social, and economic context, and truly useful digital tools will help people manage their needs in a comprehensive and integrated way rather than focus on a disease or issue in isolation. This has not been a major goal for health systems or a major focus of quality improvement efforts; however, it could be supported by consumer-facing tools that engage and empower individuals.

### Conclusion

Characterizing the needs of target users is essential to designing a comprehensive digital health advisor, but to realize this vision we need to address broader system and policy constraints that will impact its development. User-centered design can help draw attention to something that is technically feasible but has no business model and may not be compatible with many current regulations. It helps reframe problems and suggest solutions that may be independent of existing services or processes. In the area of models of care for HNHC patients, the Commonwealth Fund has commissioned rigorous evaluations of new models, synthesized evidence from the scientific literature, and created a playbook for health systems to help implement promising models and improve care for this group. We have also started using design thinking as a tool to generate a pipeline of new options that might constitute a breakthrough. We move this future-oriented work forward through our role as a convener and advocate for improving access to care. For the IT-enabled consumer, the next steps include exploring the type of collaboration needed between stakeholders such as health care delivery organizations, patients, caregivers, technology companies, government, and consumer advocacy groups. We also need to examine the incentives and business models needed to attract entrepreneurs and developers to work in this area and for health systems to engage with consumer-facing IT tools. System change is unlikely to come from those who already run it, so the Commonwealth Fund is working to address the policy and regulatory, business model, and cultural barriers to creating tools that put patients and their goals at the center of the health system.

## Appendix

Multimedia Appendix 1 Patient and caregiver personas.

## Abbreviations

HCD: human-centered design
HNHC: high-need, high-cost
ICT: information and communication technologies
IT: information technology
