# Supplementary material for: Using Human-Centered Design to Build a Digital Health Advisor for Patients With Complex Needs: Persona and Prototype Development
Source: J Med Internet Res. 2019 May 9;21(5):e10318. doi: 10.2196/10318 (PMC6532339; doi:10.2196/10318)
Supplement: Multimedia Appendix 1 [file jmir_v21i5e10318_app1.pdf]

PERSONA

Frail Elderly

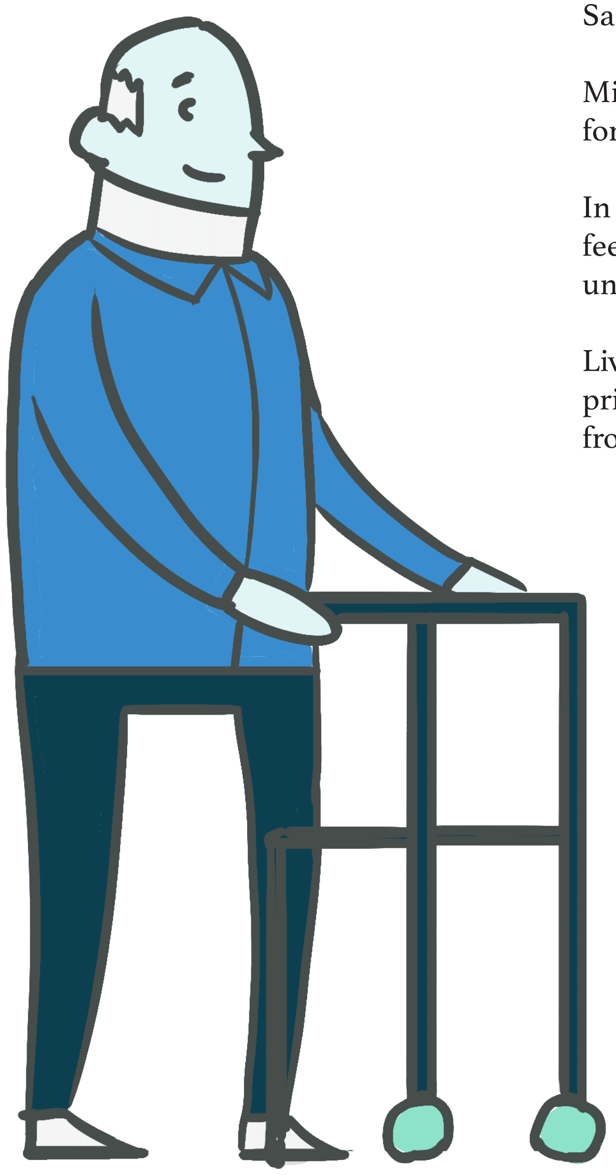

JOHN

87

San Mateo, CA

Mild arthritis, hypertension, and forgetfulness

In decent shape but recently began feeling more tired, anxious, and uncoordinated.

Lives at home with his wife as his primary Caregiver, with support from their daughter.

JOBS TO BE DONE

Even though most of his friends have passed and his family is busy, he wants to **stay active and social** so his life feels more fulfilling.

Despite his frustration that he can no longer drive, he wants to **get around and run errands** so he doesn't feel like a burden.

While his memory may be in decline, he wants to **manage his doctors appointments and retain what his doctor tells him** so he can remain capable.

Although he often forgets what his children teach him, he wants to **master his smartphone** so he can feel up-to-date and connected to his family.

Though the conversation is painful and awkward for his family, he wants to **create end of life plans** so he can have peace of mind.

A DHA CAN

Guide me and my loved ones through tough conversations.

Help me know my limitations and receive just the right amount of help.

Teach me patiently and give me opportunities to grow.

PERSONA

Caregiving Spouse

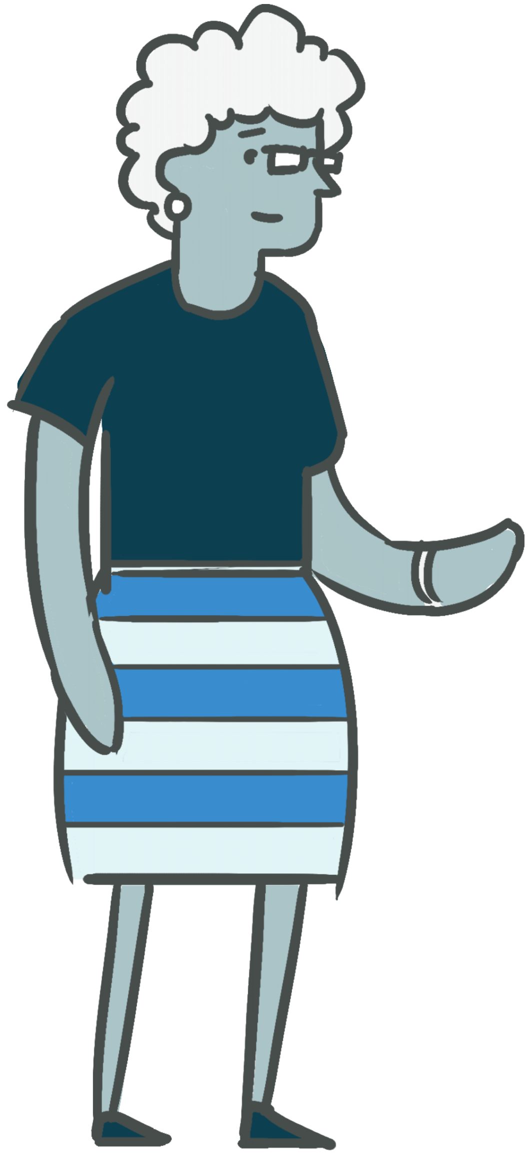

BETH

79

San Mateo, CA

Tiredness

Generally healthier and more active than her husband.

Lives at home and functions as his primary Caregiver with support from their daughter.

JOBS TO BE DONE

While she remembering to take her own, she needs to **closely manage her husband's medication** to be confident that she is providing the best care.

She needs to **find inexpensive and trustworthy resources** to help her around the house and with her husband's care to relieve her own stress.

Sometimes she doesn't know what to ask but she needs to **take notes and get answers** at doctor's appointments so she feels confident and informed.

She and her husband are overlooked in healthcare so she needs ways to **advocate on his behalf** so she knows he is getting the care he deserves.

Although he is used to relying on her she needs to **help her husband take control over his own health** so he is invested in better outcomes.

A DHA CAN

Connect me with reliable help I can trust.

Help me establish best practices and course-correct care at home.

Encourage my patient to be open and cooperative about receiving help.

PERSONA

Elderly Patient with Multiple Chronic Conditions

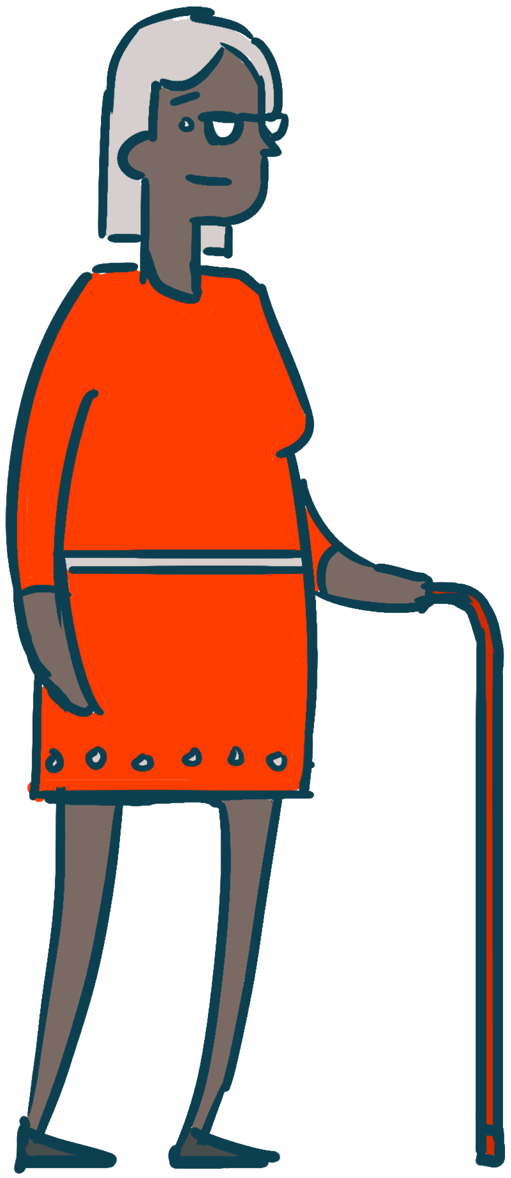

ELIZABETH

70

Long Island, NY

Grade 3 COPD, diabetes, vertigo, heart failure

Lives alone in a low-income senior apartment, near her daughter.

Needs to visit the ER several times a year for COPD attacks. On Medicaid and relies heavily on her children for support.

JOBS TO BE DONE

It's hard to know which triggers and symptoms matter but she needs to **know what changes in her condition mean** so she can make appropriate choices.

Multiple prescriptions bring the possibility of conflict, she needs to **safely manage her pill regiment** to avoid decline or hospitalization.

Some chores like cleaning and grocery shopping leave her out of breadth, she needs to **find additional help without asking more of her children.**

She is unsure if or when her condition will improve but she wants to **get back to the things she loves** like dancing to enjoy her life.

Her friends and family dismiss her feeling but she needs a way to **express her anxieties** so she can feel heard and move on.

A DHA CAN

Listen to me without making assumptions.

Guide me in the moment with clear next steps.

Help me regain the ability to enjoy my favorite things in life.

PERSONA

Caregiving Kids

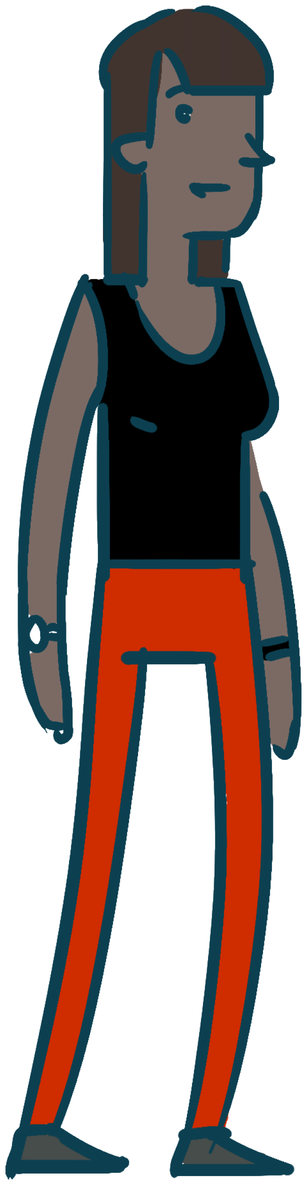

LISA

45

Long Island, NY

Married with two children (15, 18), has a part time job and provides some financial support for her mom. Usually visits 3 times a week.

Primary Caregiver for her mom with help from her two siblings.

JOBS TO BE DONE

Overcome legal and systemic barriers with insurance and doctors when trying to coordinate care on behalf of her mother.

She needs to **find inexpensive and trustworthy resources** to help her mom and relieve her own stress

Sometimes she doesn't know what to ask but she needs to **take notes and get answers** at doctor's appointments so she feels confident and informed.

She cant always be there so she needs to **share important tasks and decisions** with her siblings so mom is always covered.

Without neglecting one she needs to **balance work, her own household and errands for her mom** to keep everyone happy.

Even though her mom resists the activities she needs to **keep mom's mind sharp to prevent even more burdensome decline** or accidents.

Take appropriate action during crisis moments to limit the burden of overreacting or making uninformed decision.

A DHA CAN

Guide me in the moment with clear next steps.

Help me establish best practices and course-correct care at home.

Make it easier for authorized users to participate in my care.

PERSONA

Young Patient with Acute Chronic Condition

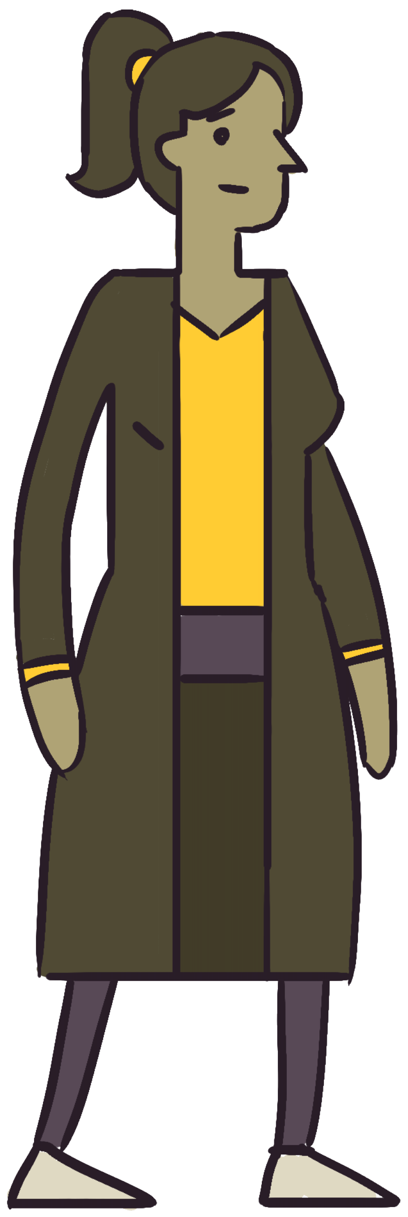

JASMINE

44

Baltimore, MD

Lupus and Depression

Lives alone and works full time. Her illness is affecting her professional and social life.

Mostly manages her own health but her primary Caregiver is her sister. She also receives support from a close friend and an online community of Lupus patients.

JOBS TO BE DONE

Without relying on medications and harsh treatments she needs to **manage symptoms and prevent flare-ups** to prevent decline.

Get as many chores done herself and **easily find additional help when she needs it** so she feels independent and capable.

Find clinical trials or new treatments that might help to **expand her care options** so progress feels more possible

Though the conversation is painful and awkward for her family, she wants to **create end of life plans** so he can have peace of mind

Her illness sometimes consumes her so she needs to **maintain her identity and nourish her passions**, like fashion and beauty

Despite her normal facade, she wants to **help other people understand her illness** so they show her more empathy.

A DHA CAN

Help me regain the ability to enjoy my favorite things in life.

Guide me in the moment with clear next steps.

Encourage empathy and understanding among my care team.

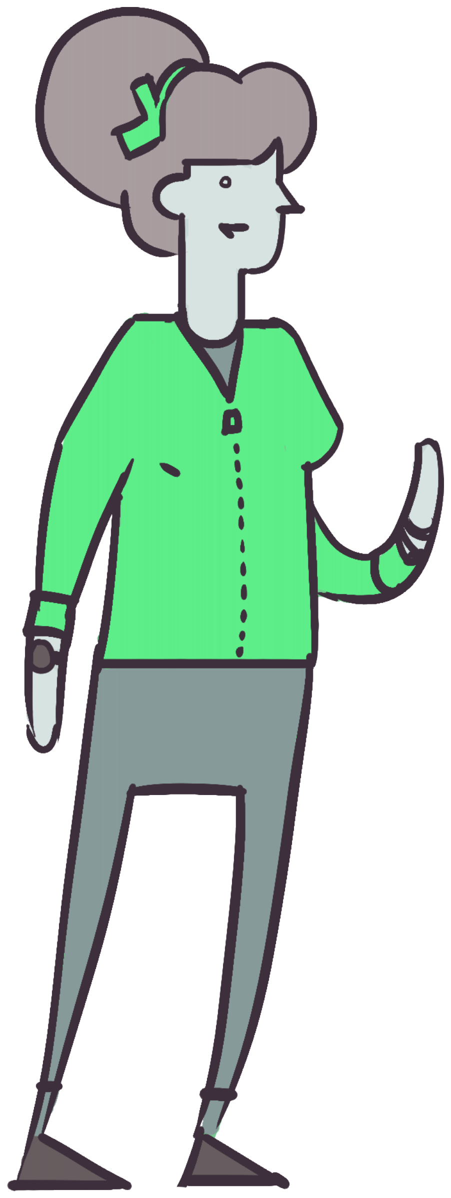

PERSONA

Patient with Multiple Chronic Conditions

KAREN

65

Baton Rouge, LA

Arthritis, Diabetes, and Asthma

Manages her own day to day health as her children live across the country. Values her independence.

Recently became eligible for Medicare.

JOBS TO BE DONE

Find the right tools and stay informed so she can **maintain a sense of control and independence** over her life.

Break through the paperwork and **find clear and easy ways to apply for services and resources** that she needs as she ages.

Multiple prescriptions bring the possibility of conflict, she needs to **safely manage her pill regiment** to avoid decline or hospitalization.

Take appropriate action during crisis moments to limit the burden of overreacting or making uninformed decision.

Give the appropriate level of attention to her diseases without letting them consume her life so she can focus on her family and passions.

She wants to track her health but needs ways to **make sense of all the numbers, levels, instructions, and advice** so she can take action.

A DHA CAN

Guide me in the moment with clear next steps.

Show me what I should do and where I stand in the process.

Help me make sense of complex data and metrics.
